# Using a Stick Does Not Necessarily Alter Judged Distances or Reachability

**DOI:** 10.1371/journal.pone.0016697

**Published:** 2011-02-24

**Authors:** Denise D. J. de Grave, Eli Brenner, Jeroen B. J. Smeets

**Affiliations:** 1 Research Institute MOVE, Faculty of Human Movement Sciences, VU University, Amsterdam, The Netherlands; 2 Unilever R&D Vlaardingen, Vlaardingen, The Netherlands; University of Regensburg, Germany

## Abstract

**Background:**

It has been reported that participants judge an object to be closer after a stick has been used to touch it than after touching it with the hand. In this study we try to find out why this is so.

**Methodology:**

We showed six participants a cylindrical object on a table. On separate trials (randomly intermixed) participants either estimated verbally how far the object is from their body or they touched a remembered location. Touching was done either with the hand or with a stick (in separate blocks). In three different sessions, participants touched either the object location or the location halfway to the object location. Verbal judgments were given either in centimeters or in terms of whether the object would be reachable with the hand. No differences in verbal distance judgments or touching responses were found between the blocks in which the stick or the hand was used.

**Conclusion:**

Instead of finding out why the judged distance changes when using a tool, we found that using a stick does not necessarily alter judged distances or judgments about the reachability of objects.

## Introduction

There have been frequent reports that perception of action goals is affected by the possibilities to act upon the goal. For example, hills look steeper when wearing a heavy backpack [1] (but see [2], [3]), children perceive a target to be larger after hitting it with a ball [4], a baseball is perceived to be larger after it has been hit successfully [5], darters estimate the target to be larger when they are more accurate [6] and golfers perceive the hole to be larger after a successful putt [7]. When using a tool to perform an action, the perceived distance of the goal object can also be affected. An object is judged to be closer when a stick is used to touch the object's remembered location than when touching is performed with the hand [8].

It is unclear what kind of re-calibration takes place in the above-mentioned studies. For the effect of tool use on distance perception, one can think of at least two explanations. The first one is that distances are judged in relation to affordances such as reachability, so that a tool that expands the range of positions that are reachable stretches the ruler which is used to scale apparent distance, leading to a decrease in the judged distance of the target [9]. Alternatively, tool use might expand the representation of the participant's limb so that it encompasses the tool [10]–[12]. If the fact that the representation encompasses the tool is ignored in subsequent judgments, then the expanded representation could be considered as an increase in the judged length of the arm (although arm length is not judged as such). Using a measure related to arm length as a reference for distance judgments could also result in closer distance judgments after tool use.

To distinguish between the explanation that visual space is scaled and the one that judged arm length is extended, we perform three sessions of estimation trials interleaved with touching trials. In the first session there are trials in which participants are asked to estimate the distance of an object (in cm) interleaved with trials in which they are asked to touch the remembered location of the object (with and without a tool). This session is a replication of the first experiment in the study of Witt et al. [8].

**Figure 1:**
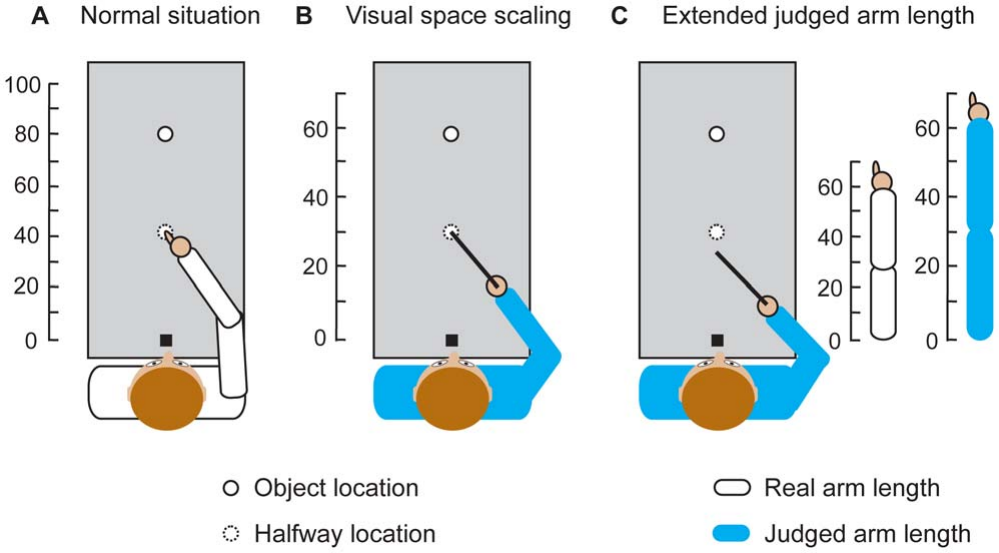
Explanations for the effect of tool use on perceived distance. Expected touch locations in session 2 in the normal situation (A), for the visual space scaling explanation (B) and the explanation based on extended judged arm length (C).

In the second session we investigate whether the perceptual effect would also appear in the performed action. The data from the first session cannot provide an answer to this question because participants cannot touch the object in many trials. Therefore, participants performed the same estimation task, but now, in the touching trials, we asked the participants to touch halfway between the remembered object location and the start position (figure 1A). According to the visual space scaling explanation participants use a stretched ruler to estimate the distance of the object. Irrespective of where the object is located along this ruler, participants will touch the location halfway between the object and the start position (figure 1B). Thus no effect on touching is expected. On the other hand, according to the explanation based on extension of judged arm length there should be an effect on the touching trials. In this explanation participants scale distances to their arm length. If judged arm length is increased due to tool use, objects are perceived closer to the body. Therefore participants think they don't have to stretch out their arm as much. This results in touching closer than the location halfway between the object and the start position (figure 1C).

Finally in the estimation trials of the third session participants are asked to judge whether an object can be reached. More specifically, we asked them whether the target could be touched with the hand (without actually doing so). This too was done in order to distinguish between the visual space scaling explanation and the explanation based on extended judged arm length. The visual space scaling explanation does not predict an effect of tool use on reachability judgments, whereas the extended judged arm length explanation predicts an increase in the boundary of estimated reachability after tool use. These estimation trials are interleaved with touching halfway trials, as in the second session.

The data show no differences between distance estimations (session 1 and 2) or reachability judgments (session 3) when touching with the hand or with the stick. No effect of tool use was found in the performed action either (session 2 and 3). Thus, instead of finding out why the judged distance changes when using a tool, we found that using a stick does not necessarily alter judged distances or judgments about the reachability of objects.

## Methods

### Ethics Statement

On the sixth of February 2006, the Ethical Committee of the Faculty of Human Movement Sciences expressed the opinion that there were no ethical objections against conducting the research described in the program entitled “Human Perception and Motor Control”. The current study is part of that program and has been conducted in accordance with the principles of the Helsinki 1964 declaration.

### Participants

Six participants took part in the three sessions of the experiment. All participants had normal or corrected-to-normal vision and were right-handed by self-report. They all gave their written informed consent prior to their inclusion in the study.

### Stimuli and apparatus

**Figure 2:**
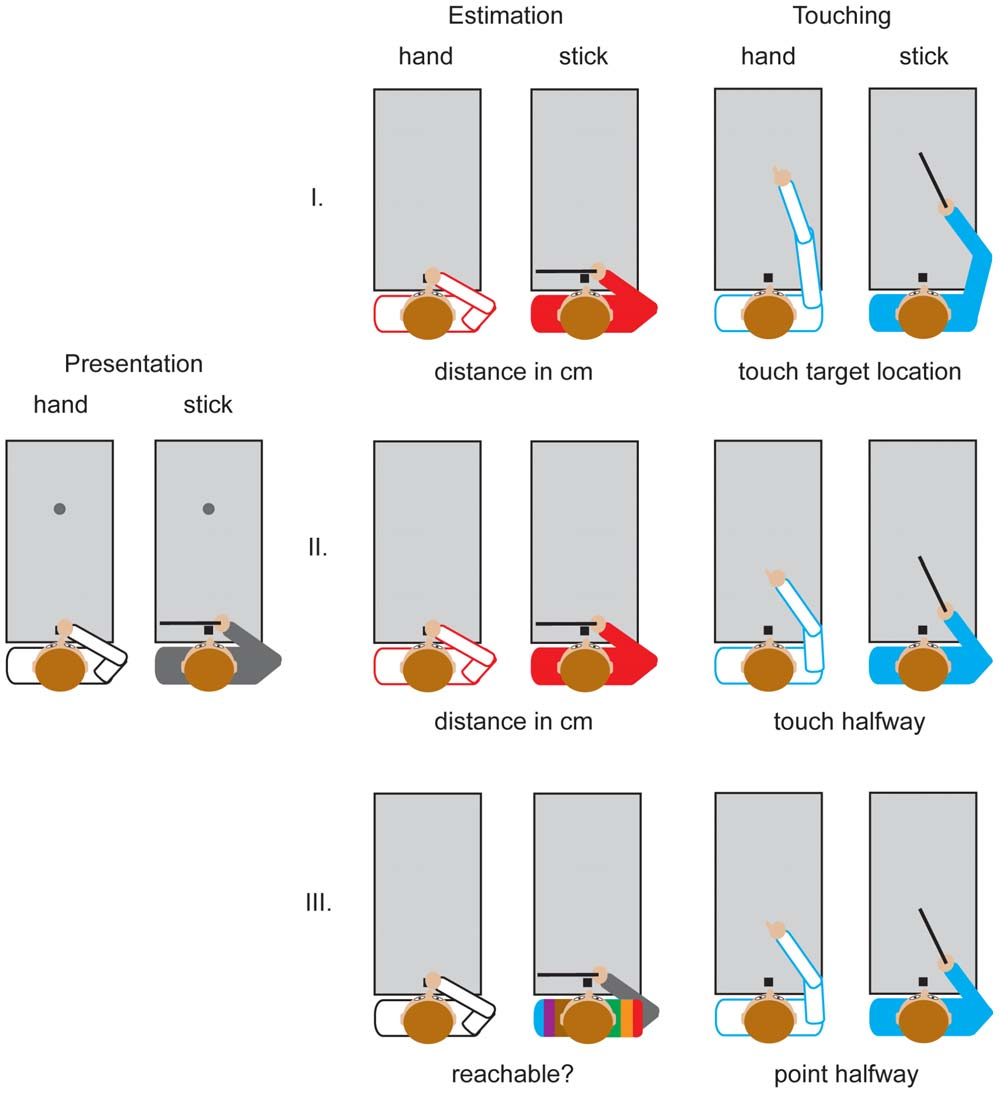
Top view of the experimental set-up for the three sessions. First participants are shown an object on the table. Then, depending on the session, they either estimate the distance of the object or determine whether the object is reachable with their hand. Estimation trials are randomly interleaved with touching trials. Whether touching is done with the hand or with the stick is blocked. Depending on the session, participants either touch the remembered location of the object or touch the location halfway between the object and the starting position.

Participants sat in front of a rectangular table (160×80 cm) with their upper body against the table, which prevented them from bending over the table. Their body midline was aligned with a start position (figure 2). The start position was a small wooden block (2.5×2.5×2.5 cm) with its nearest edge 6 cm from the nearest edge of the table. The start position specified the location from which the distance to the stimulus object was to be estimated (session 1 and 2). The stimulus object consisted of a white plastic cylinder (diameter: 5 cm; height: 10 cm). The table-top was uniformly green to minimize landmarks that could influence distance judgments. The table was in a typically cluttered laboratory environment, but there were no objects in the space immediately surrounding the table. Each session contained two blocks of trials. In one block of trials participants used a wooden stick (length: 55 cm) to perform a touching task. A marker was attached to the distal end of the stick to track its movement with an Optotrak 3020 system (sampling rate 250 Hz, resolution 0.01 mm). In the other block of trials participants did not use the stick and a marker was attached to the nail of the participant's right index finger. Participants wore liquid crystal shutter glasses (Plato System; Translucent Technologies, Toronto, Ontario, Canada) so that we could control the time that the object was visible.

### Procedure

Within each session, half the participants used the stick in the first block, and the other half used the stick in the second block. In half the trials of each block participants performed an estimation task and in the other half they performed a touching task (with either hand or stick). Estimation and touching trials were randomly interleaved in a block. In the stick block participants constantly held the stick in their hand, irrespective of whether they had to touch or estimate on that trial.

At the beginning of each trial, participants held their right hand on (in the hand block) or near (in the stick block) the start position (figure 2). The shutter glasses turned opaque and the experimenter placed the object on the table. Then the shutter glasses became transparent for 1 second. When the shutter glasses turned opaque again a tone sounded for 300 ms to identify the task and the experimenter removed the object from the table. The pitch of the tone indicated the participant's task (1000 Hz for estimation; 400 Hz for touching). In the estimation task of sessions 1 and 2, participants had to estimate the distance of the object from the start position (in cm). In the estimation task of session 3, participants had to judge whether they would be able to reach the object with their hand, without trying to do so. During all estimation tasks the shutter glasses remained opaque. When the task was to touch (either with their hand or with the stick), the shutter glasses became transparent again for 3 seconds (2 seconds after they had been closed). In session 1 participants were instructed to touch the location of the object. If the location previously occupied by the object was within reach, participants reached out and touched it. If it was beyond reach, participants pointed to where it had been without placing their hand on the table. In sessions 2 and 3, the task was to touch the location (on the table) halfway between the object and the start position.

Participants did not know whether they would have to touch or estimate until after view of the object was blocked. This was done in order to prevent them from attending to different sources of information depending on the task [13]. Moreover, in this way we ensured that participants were aware in all trials that the tool increased their reaching span. They were not given prior training and no feedback was given on touching or estimating accuracy. Session 1 and 2 each contained 180 trials: 2 blocks (hand or stick)×2 tasks (estimation or touching)×9 predefined object distances (50, 57, 63, 68, 75, 83, 91, 96, 99 cm in session 1 and 45, 50, 54, 59, 63, 68, 73, 80, 90 cm in session 2)×5 repetitions. The duration of each session was about one hour. Participants were not told the range of distances that would be used and were not given a source with which to calibrate their estimates. In session 3, each block contained 2 staircases for the estimation trials (one ascending and one descending). The ascending staircase started at an object distance of 58 cm and the descending staircase started at 90 cm. If judged reachable, the location of the object in the next trial was shifted toward the participant by 3 cm in the descending staircase and by 2 cm in the ascending staircase. If judged not to be reachable, the object was shifted away from the participant by 2 cm in the descending staircase and by 3 cm in the ascending staircase. The object distances of the staircases of the estimation trials were also used for the touching trials. The total number of trials in session 3 was 160 (80 estimation trials [each staircase of estimation trials contained 20 trials] and the same number of touching trials). Session 3 took about 45 minutes.

### Data analysis

For the estimation trials in session 1 and 2 we calculated the average estimated values for each block, object distance and subject. The average values for the two blocks (hand, stick) are compared with t-tests. For the estimation trials in session 3, we calculated for each subject the proportion of “no” answers at each presented object position (ascending and descending staircase taken together). Psychometric functions (cumulative normal distributions) were fitted for each participant and each block using the Matlab psignifit toolbox version 2.5.6 which implements the maximum-likelihood method described by Wichmann and Hill [14]. The fitted parameters for the sigmas and the 50% value (boundary of reachability) were compared with paired t-tests.

For the touch trials in each session we calculated the average distance between the touched location and the start position for each block, object distance and subject. In session 1 object distances at which participants pointed to the perceived object location (instead of touching it) on 50% or more of the trials were excluded from analysis. Average distances for the blocks in each session are compared with t-tests.

## Results

### Session 1

**Figure 3:**
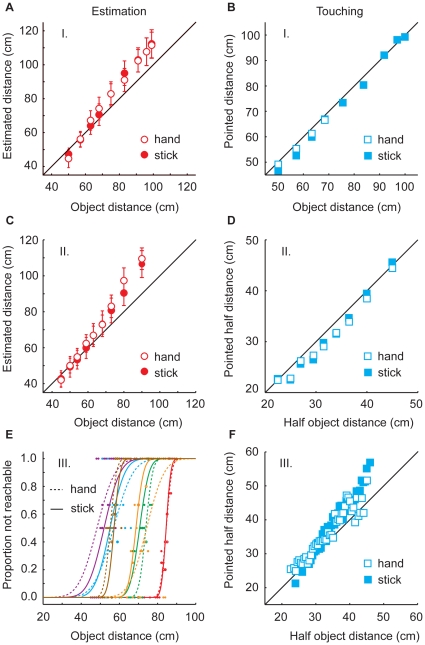
Data of the estimation and touching trials in the three sessions. Session 1 (A, B), session 2 (C, D) and session 3 (E, F). Open symbols and dotted lines represent data from the hand block and filled symbols and solid lines show the data from the stick block. Each color in figure 3E represents one subject. Error bars represent between-subject standard errors.

Figure 3A shows the data of the estimation trials in the *hand* block (open symbols) and the *stick* block (filled symbols). No significant difference was found between distance estimations in both blocks (t = 0.02; p = 0.97). On average, participants overestimated the distance of the object in both blocks by about 6.2 cm. The overestimation increased with object distance (slope 1.33). On average the overestimation did not differ significantly from zero. A slope larger than 1 shows that the range of estimated distances is larger than the true range of distance values. This is consistent with an error in the distance scale. No difference was found between the hand and the stick block in the touch trials either (t = −0.52; p = 0.63). Participants were fairly accurate both when they touched the object location with the stick and when they touched it with their finger (slope 1.02; figure 3B). On average there was a slight underestimation (1.6 cm), but this did not differ significantly from zero. Note that touching trials with the hand are only available for the near half of the objects.

### Session 2

As for session 1, no significant difference was found between distance estimations in the hand block and those in the stick block (t = 0.68; p = 0.52). On average, participants overestimated the distance of the object by about 6.3 cm (figure 3C). The overestimation increased with object distance (slope 1.47). On average the overestimation did not differ significantly from zero. As in the first session, participants used a stretched scale for distance estimation. For the touch trials a similar pattern was found as in session 1. No significant difference was found between touching with the hand or touching with the stick (t = 0.35, p = 0.74). Participants touched on average 1.8 cm too short (figure 3D, slope 1.03). This did not differ significantly from the positions halfway between the object and the start position.

### Session 3

Figure 3E shows the psychometric curves for reachability estimates of each subject in both the hand and the stick block. Neither the 50% values nor the sigmas of the individual participants' psychometric curves showed a significant difference between the hand and the stick block. The average difference in estimated reachable distance between the two blocks was 0.9 cm, with a slightly larger value for estimations in the hand block (one-sided t-test: t = −0.60, p = 0.71). The average sigma was 4.6 cm. In this session, again no difference was found between touching with the hand and touching with the stick (t = −0.60, p = 0.57; figure 3F). On average, participants touched 3.3 cm too far (slope 1.34), which was not different from the positions halfway between the object and the start position.

## Discussion

In this study we investigated whether changed judgments of objects' distances after tool use can be ascribed to a scaling of the reachable range or to changed judgments of arm length. Unfortunately, we cannot conclude which one of these two explanations can best describe the results found in previous studies (e.g., [8]), since we did not find any differences in estimated distances or judged reachability between the block in which participants had to use the stick for touching and the one in which the hand had to be used. Furthermore, no differences in touched locations were found when touching movements were made with the hand or the stick.

As previously found for effort-related effects on perceived distance, our data suggests that the influence of tool use on estimated distance is more fragile than has heretofore been appreciated [3], [15], [16]. Small differences in experimental design might be of vital importance. In our study the tool was used on randomly selected trials, as opposed to on every trial in the study of Witt & Proffitt [17]. However, this is unlikely to be the explanation of the difference, because in an earlier study of Witt et al. [8] participants also did not use the tool on each trial (participants either had to touch or estimate, as in the present study), and nevertheless an effect of tool use on distance estimation was found.

In the study of Witt et al. [8] it was shown that targets only appear to be closer when participants actively use a tool. When participants never intended to reach with the tool, the perceived distance to the targets was unaffected by whether or not the tool was held. In session 1 of the current study participants reached to where the object had been, but in sessions 2 and 3 they did not reach to the object itself but reached halfway to the object. Nevertheless there was a clear intent to use the tool in a manner that was related to the judged object distance in all three sessions. Moreover, even without the action the intention to act can elicit action-specific influences on perception [4], [17]–[19] although it is not clear from previous studies whether the critical factor is the intention to use the tool or the intention to act on the object of which the distance is to be judged, and Durgin et al. [2] have even proposed that intent may not be the critical factor at all.

Effects of tool use are known to persist for some time after the tool has been used, with estimates varying from several minutes to over an hour [20]–[24]. Although in our study participants did not know whether they would use the tool on the next trial (to touch the remembered object location in session 1 or to touch the halfway location in sessions 2 and 3), they did constantly hold the tool and they always had to be prepared to use the tool, so the intention to use the tool was clearly present. Thus our failure to find an influence of holding the stick is unlikely to be related to issues related with the time between the judgments and the (intentional) use of the tool. Further studies are needed to establish what experimental or procedural manipulation (e.g. stimulus presentation time, availability of visual feedback during task performance or instructions to the participants) determines whether an effect of tool use is present.

In contrast to studies that have suggested that tool use expands the representation of the participant's limb so that it encompasses the entire tool [10]–[12] or the end-effector [23], [25], [26], Povinelli et al. [27] suggest that separate spatial representations exist for the hand and the tool in peripersonal space. This could explain the absence of a tool effect in the touching trials of our study, because participants would use the appropriate representation to determine the location of the object for each task. That would be consistent with the touched positions being similar for the tool and the hand block in sessions 2 and 3 and for participants not attempting to reach the far objects when not using the stick in session 1. Overall, we conclude that using a stick does not necessarily alter judged distances or the judged reachability of objects.
